# Cepstral Analysis for Scoring the Quality of Electrocardiograms for Heart Rate Variability

**DOI:** 10.3389/fphys.2022.921210

**Published:** 2022-06-17

**Authors:** Paolo Castiglioni, Gianfranco Parati, Andrea Faini

**Affiliations:** ^1^ IRCCS Fondazione Don Carlo Gnocchi ONLUS, Milan, Italy; ^2^ IRCCS Istituto Auxologico Italiano, San Luca Hospital, Milan, Italy; ^3^ Department of Medicine and Surgery, University of Milano-Bicocca, Milan, Ital

**Keywords:** heart rate variability, fourier transform, EKG, power cepstrum, signal quality, premature beat, wearable system, mobile ECG monitoring

## Abstract

Mobile-health solutions based on heart rate variability often require electrocardiogram (ECG) recordings by inexperienced operators or real-time automatic analyses of long-term recordings by wearable devices in free-moving individuals. In this context, it is useful to associate a quality index with the ECG, scoring the adequacy of the recording for heart rate variability to identify noise or arrhythmias. Therefore, this work aims to propose and validate a computational method for assessing the adequacy of single-lead ECGs for heart rate variability analysis that may run in real time on wearable systems with low computational power. The method quantifies the ECG pseudo-periodic structure employing cepstral analysis. The cepstrum (spectrum of log-spectrum) is estimated on a running ECG window of 10 s before and after “liftering” (filtering in the cepstral domain) to remove slower noise components. The ECG periodicity generates a dominant peak in the liftered cepstrum at the “quefrency” of the mean cardiac interval. The Cepstral Quality Index (CQI) is the ratio between the cepstral-peak power and the total power of the unliftered cepstrum. Noises and arrhythmias reduce the relative power of the cepstral peak decreasing CQI. We analyzed a public dataset of 6072 single-lead ECGs manually classified in normal rhythm or inadequate for heart rate variability analysis because of noise or atrial fibrillation, and the CQI = 47% cut-off identified the inadequate recordings with 79% sensitivity and 85% specificity. We showed that the performance is independent of the lead considering a public dataset of 1,000 12-lead recordings with quality classified as “acceptable” or “unacceptable” by visual inspection. Thus, the cepstrum describes the ECG periodic structure effectively and concisely and CQI appears to be a robust score of the adequacy of ECG recording for heart rate variability analysis, evaluable in real-time on wearable devices.

## 1 Introduction

Advancements in sensors technology are making it possible to monitor the electrocardiogram (ECG) for long periods in unattended subjects through wearable systems, promoting solutions for telemonitoring, home rehabilitation, mobile health, and ambient-assisted living applications. Most of these applications quantify indexes of heart rate variability to provide information on the autonomic control and cardiorespiratory interactions, based on ECG recordings performed by inexperienced users and on automatic analyses of ECG tracings. In these cases, it is important to associate a quality score with the recorded signals. In telemonitoring applications this would indicate to inexperienced operators the need to repeat the recording if the ECG quality is too low; in ambient assisted living applications, this would allow expert systems not to take decisions on the base of unreliable ECG signals. A further requirement is to exclude arrhythmias if the aim is to quantify heart rate variability, as during exercise-based rehabilitation programs for restoring the autonomic control in cardiac patients after heart surgery or in diabetic individuals with autonomic neuropathy. A normal rhythm is indeed necessary to correctly interpret the indices of heart rate variability.

In the frame of domotic applications aimed at developing a smart environment for elderly people, we had to deal with the definition of an automatic score of the ECG quality in normal rhythm. The domotic application consisted of a first layer of sensors and devices with low computational power to collect physiological and behavioral data to be sent to upper computational levels operating decisions in support of the assistance staff (Gower et al., 2011). In particular, the ECG had to be recorded for hours or days on freely moving subjects for a continuous assessment of heart rate variability with wearable ECG sensors (Di Rienzo et al., 2010). In this context, the occurrence of arrhythmic episodes, artifacts, and noise was expected, making important the dynamic assessment of data reliability in real-time automatically. These requirements demanded an algorithm working on different leads separately and running on low-power microprocessors on board the wearable devices, to score the ECG quality, select the best lead, and identify the presence of normal sinus rhythm for the online evaluation of heart rate variability. To deal with these requirements, we originally designed an algorithm to characterize the ECG quality from its periodic structure (Castiglioni et al., 2011).

We further developed the original algorithm and this work aims to illustrate the capability of cepstral analysis to characterize the pseudo-periodicity of the ECG and to propose and validate a cepstral method for devices with low-computing power that scores the quality of ECG leads for heart rate variability applications.

## 2 Methods

### 2.1 The Power Cepstrum

The Power Spectrum *PS(f)* of a signal *s(t)* is the squared magnitude of its Fourier Transform:

$$PS\left(f\right)={\left|ℱ\left[s\left(t\right)\right]\right|}^{2}$$ (1)

The Power Cepstrum *PC(*τ*)* of *s(t)* is the power spectrum of the logarithm of *PS(f)*:

$$PC\left(\text{τ}\right)={\left|ℱ\left[\log PS\left(f\right)\right]\right|}^{2}$$ (2)

The Power Cepstrum was introduced to identify signals echoes (Bogert et al., 1963; Oppenheim and Schafer, 2004). In fact, the Fourier spectrum of the superposition of a signal and its echo after *τ* seconds is the product between the spectrum of the signal and a periodic function with period 1/*τ* Hertz. The logarithm converts the product into a sum and the following Fourier analysis identifies the echo delay as a spectral peak at “frequency” *τ*. The domain of the spectrum of the log-spectrum is treated as a “frequency domain”, but after two Fourier transforms its units are those of time, in seconds, not of frequency, in Hertz. For this reason, it is referred to as the “quefrency” domain. Like “cepstrum” and “liftering”, this term was coined by interchanging consonants of familiar words (“frequency”, “spectrum” and “filtering”) to emphasize that time-domain methods are applied to functions of the frequency.

**FIGURE 1:**
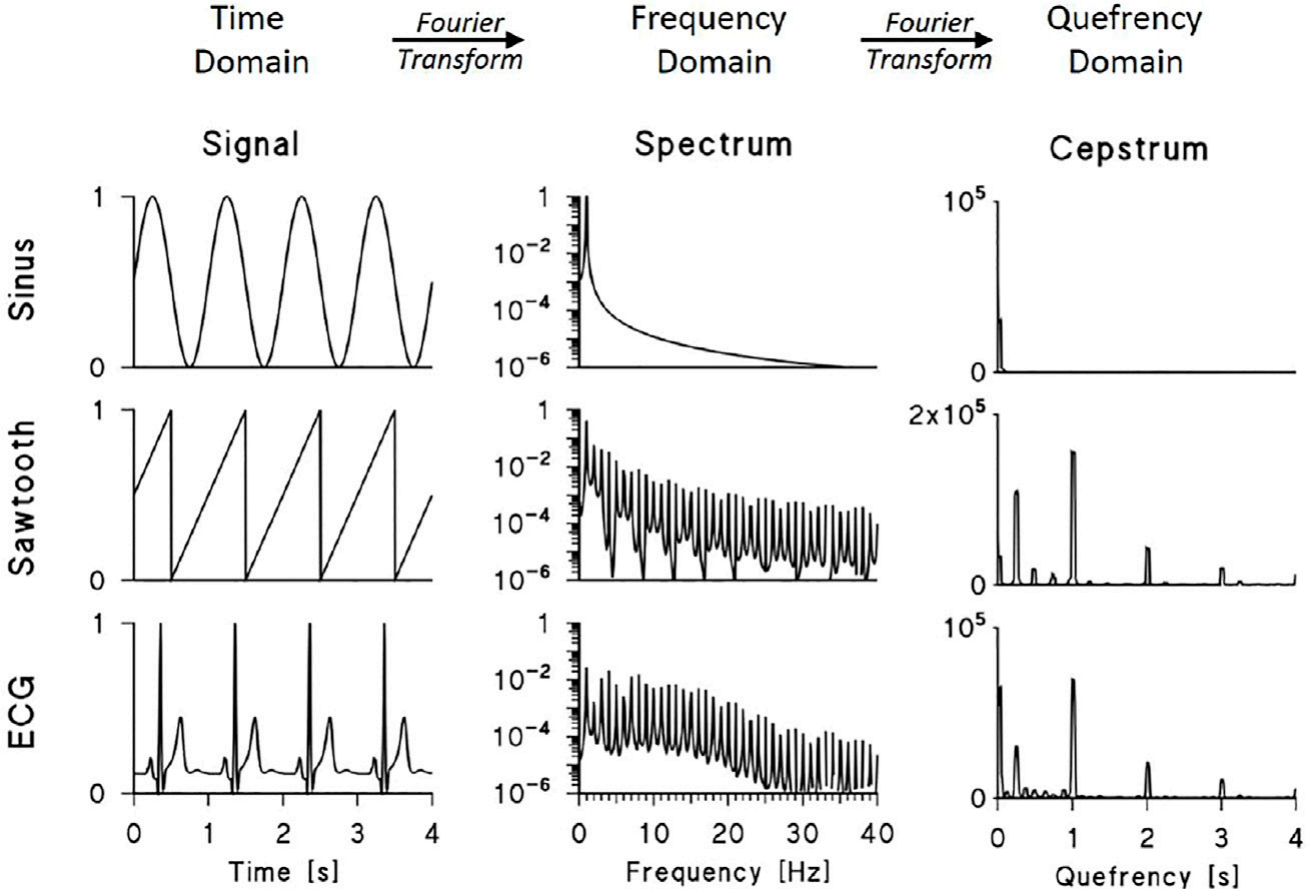
Power spectra and power cepstra of a sinusoid, sawtooth function and synthesised ECG. The signals were sampled at 200 Hz, the spectra were calculated over 2048 points by Fast Fourier Transform (FFT) and the cepstra were calculated by FFT of the log-transformed spectra between 0 and 100 Hz (for clarity, spectra are plotted up to 40 Hz, signals and cepstra up to 4 s). The cepstra of the sawtooth function and synthesized ECG show peaks at 1, 2, and 3 s representing the train of power spectrum harmonics multiple of the fundamental frequency *f*_*0*_ = 1 Hz; the sawtooth cepstrum also shows peaks at 0.24 s and its multiples representing the modulation with period of 4.2 Hz visible in its log-spectrum; the ECG cepstrum shows a large peak at the lowest quefrency representing the decreasing spectral trend.

The cepstral approach, however, also gives us a concise way of describing the harmonic structure of periodic signals. This is illustrated in Figure 1, which compares power spectra and power cepstra of three periodic functions with a period of 1 s.

The first function is a sinusoid: its power spectrum is composed of a single peak at 1 Hz and, consequently, its cepstrum shows very-low quefrency power only. The second signal is a sawtooth function: the power spectrum consists of a sequence of harmonics at multiples of the fundamental frequency *f*_*0*_ = 1 Hz. When plotted in a log scale, this train of peaks appears modulated by a slower oscillation with a “period” of 4.2 Hz. These components are clear in the cepstrum: harmonics at quefrencies *τ* multiples of *τ*_0_ = 1/*f*_*0*_, i.e. at *τ* equal to 1, 2, and 3 s, represent the train of spectral peaks; and cepstral peaks at *τ* = 1/4.2 Hz^−1^ (i.e., 0.24 s) and its multiples represent the slower spectral periodicity. The third periodic function is a synthesized ECG: in this case, the log-spectrum appears as a quasi-sinusoidal train of peaks that decays at frequencies higher than 20 Hz. Like the sawtooth cepstrum, the ECG cepstrum shows peaks at *τ* = 1, 2, and 3 s that represent the train of harmonics.

### 2.2 Synthesized Electrocardiogram

To identify the parameters that better describe the cepstral peaks of the ECG, we made use of synthesized ECG signals. The same synthesized ECGs also allow us to quantify the effects of added noise and deviations from pseudo-periodicity. To synthesize ECG waves with realistic shapes, we started with real recordings. One lead ECG (Einthoven II lead, 200 Hz) was recorded in eight young volunteers (4 males/4 females, age 21–38 years) resting supine for 10 min by a Cardioline Delta 1 plus (REMCO ITALIA, Milan, Italy) electrocardiograph. An ECG template was obtained from each recording by R-peak synchronized average. About 600 beats were averaged for each template, virtually removing any type of noise asynchronous with the R peak (baseline drift, muscular noise, or 50/60 Hz power noise). A synthesized ECG was generated from each template sequentially appending copies of the template, spaced evenly. The mean R-R interval of the 8 recordings ranged between 738 and 1126 ms and the distance between consecutive R peaks was equal to the mean R-R interval of each original recording. In this way, the 8 synthesized signals preserved the original heart rate and ECG shape (see an example in the lower-left panel of Figure 1).

### 2.3 Electrocardiogram Cepstral Estimator

We first introduced the ECG cepstral analysis in a conference presentation as a new tool for assessing the quality of electrocardiographic recordings (Castiglioni et al., 2011). In the present work, we evaluate critically the performance of the cepstral approach by applying it to synthesized ECG signals, to a large number of real ECG recordings from public databases, and to specific ECG tracings selected from our previous works as being representative of specific physiological or pathological conditions. However, before applying the cepstral analyses on synthesized and real ECGs, this paragraph shows how we empirically optimized some parameters of the cepstral estimator. In particular, the power cepstrum estimator depends on the type of data windowing and spectral smoothing, like the traditional Fourier periodogram. Since the cepstrum consists of two consecutive Fourier spectra, the length of the data windows should be defined in the time domain for the first Fourier Transform, selecting the duration of the ECG segments, and in the frequency domain for the second Fourier Transform, selecting the frequency range of the log-spectrum. The window length in the time domain was set equal to 10 s as a trade-off between frequency resolution, which should be sufficiently high to distinguish ECG harmonics, and amplitude of heart rate changes, which should be relatively small to locally preserve the pseudo-periodicity of the signal.

**FIGURE 2:**
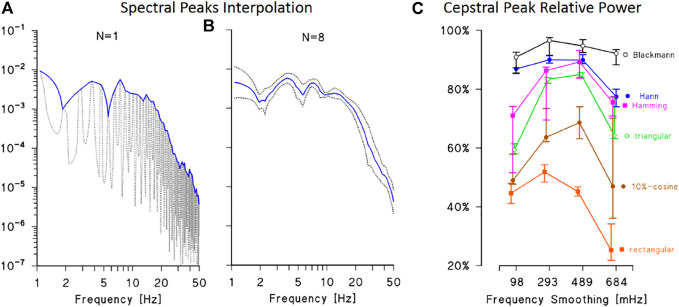
Definition of data windows for ECG cepstral analysis. **(A)**: power spectrum of a synthesised ECG (dotted line) with interpolation of the maxima at the fundamental harmonic and its multiples (continuous line). **(B)**: average and 95% confidence interval of the lines interpolating the spectral maxima of 8 synthesised ECG. The average interpolating line is relatively constant up to 20 Hz and rapidly decreases at higher frequencies, suggesting to limit the length of the frequency-domain data window up to 20 Hz. **(C)**: relative power of the main cepstral peak: median and interquartile range over 8 synthesised ECGs calculated by six different time-domain data windows and by smoothing the spectral lines using moving average filters with bandwidth between 98 and 684 mHz. The best performance is obtained with the Blackmann window and smoothing bandwidth between 250 and 500 mHz.

The optimal window length in the frequency domain was identified as the frequency band where the height of ECG spectral peaks remains relatively constant. This choice avoids introducing very low quefrency components in the cepstrum due to slow decreasing trends in the train of harmonics. The band was identified by calculating the power spectrum for consecutive 10-s segments of each synthesized ECG and by interpolating the maxima (Figures 2A,B). The interpolating function is relatively stable below 20 Hz and decreases at higher frequencies, coherently with the literature (Golden et al., 1973). This suggests setting the length of frequency-domain data windows between 0 and 20 Hz.

The type of window function critically defines the proper combination between power leakage and main-lobe amplitude of the Fourier spectrum (Marple, 1987), determining the relative power of the cepstral peak at the quefrency *τ*_0_ corresponding to the mean R-R interval. To choose the time-domain data window, the relative amplitude of the main cepstral peak was calculated using six different windows (rectangular, triangular, 10%-cosine, Hann, Hamming, and Blackman) and by smoothing the resulting spectral lines with moving average filters of different orders. The best performance was obtained with the Blackmann window (Figure 2C): about 90% of the cepstral power is concentrated in the main cepstral peak if the Blackman window is used in the time domain and the resulting spectral lines are smoothed with moving average filters of order between 250 and 500 mHz.

As regards the data window in the frequency domain, we selected the 10%-cosine taper (Bingham et al., 1967) because the window length is relatively short (up to 20 Hz only, implying a relatively large main lobe) and the cosine taper reduces leakage with a small increase only of the width of the main lobe.

### 2.4 A Cepstral Score of the Electrocardiogram Quality in Normal Sinus Rhythm

The evidence that the harmonic structure of synthesized ECGs produces cepstra with a dominant peak at the quefrency *τ*_0_ corresponding to the average R-R interval, suggests taking the relative power of this cepstral peak (and its higher harmonics if present) as the index of ECG signal quality. To obtain a Cepstral Quality Index (CQI), first the ECG cepstrum and its total power are estimated as follows:

1. a 10-s ECG segment is selected, linearly detrended and Blackman windowed, *s(t)*;
2. the FFT power spectrum of *s(t)* is calculated, *P(f)*;
3. *P(f)* is truncated at 20 Hz, smoothed averaging contiguous lines over a frequency band of 300 mHz and log-transformed, log *P(f)*;
4. log *P(f)* is linearly detrended by least-square fitting a regression line and windowed by the 10%-cosine taper, log *P(f)*_*CT*_;
5. the FFT power spectrum of log *P(f)*_*CT*_ is calculated obtaining the cepstrum *CP(τ)* at quefrencies *τ≥* 0.05 s (inverse of 20 Hz, highest frequency in the log spectrum);
6. the total cepstral power TOT is calculated by integrating *CP(τ)* up to *τ* = 3 s.

**FIGURE 3:**
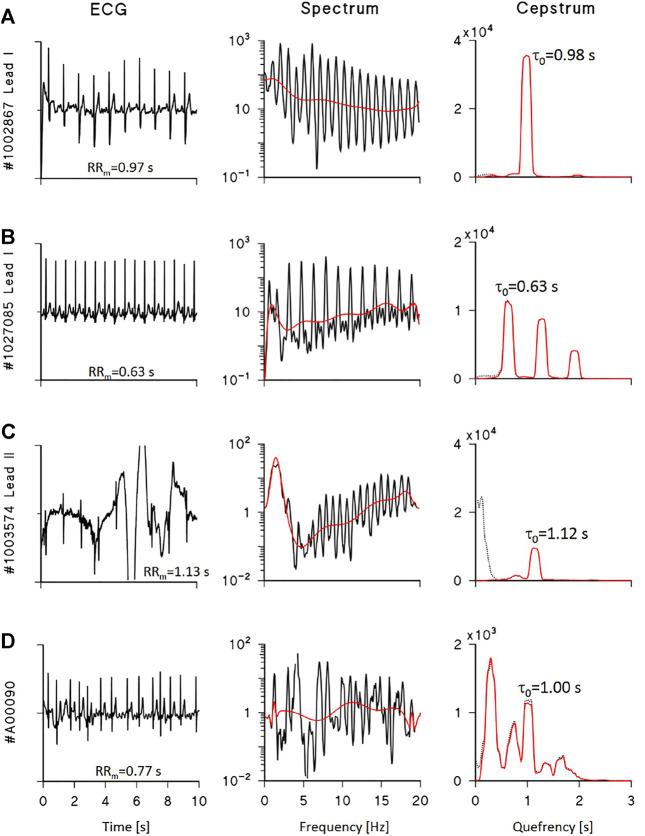
Examples of ECG cepstral analysis. *Left*: 10-s ECG segments from the Physionet/Computing in Cardiology datasets of the 2011 **(A–C)** and 2017 **(D)** Challenges; *centre*: log-spectra with polynomial trends (red line); *right*: cepstra before (dotted black) and after (red) liftering by polynomial detrending, with *τ*_0_ the quefrency of the estimated first harmonic. **(A)** shows a high quality ECG and a cepstrum with a single harmonic; the corresponding CQI is 91.1%. **(B)** shows a high quality ECG, its cepstrum with 3 harmonics multiple of *τ*_0_, and CQI is 67.2%. **(C)** shows a low-quality ECG with a single harmonic identifiable in the liftered cepstrum and CQI is 29.3% due to consistent low-quefrency noise. **(D)** shows a high quality ECG during atrial fibrillation: the log-spectrum does not have a periodic structure, the liftered cepstrum does not show a clear first harmonic, and CQI is 22.1% only. When a clear cepstral first harmonic is identifiable **(A–C)**, its quefrency *τ*_0_ practically coincides with the mean R-R interval, RR_m_.

Figure 3 shows examples of power cepstra *CP(τ)* from real ECG recordings selected from the datasets of the PhysioNet/Computing in Cardiology 2011 Challenge (Silva et al., 2011) and 2017 Challenge (Clifford et al., 2017). Figures 3A,B show high-quality ECG in normal sinus rhythm: their cepstrum consists of the main harmonic at the quefrency of the mean R-R interval and possibly higher harmonics. By contrast, Figure 3C shows that noise may produce spurious cepstral peaks with larger power than the true ECG peak; in this case, high-pass “liftering” (i.e., filtering in the frequency domain) the log-spectra may help identifying the true peaks. Thus, to properly calculate the power associated with the ECG cepstral harmonics, a liftered cepstrum is also estimated as follows:

7. log *P(f)* (calculated at step 3) is “liftered” by least-square fitting and removing a polynomial of order 10, log *P(f)*_*L*_;
8. the 10% cosine taper is applied to log *P(f)*_*L*_ obtaining log *P(f)*_*LCT*_;
9. the FFT of log *P(f)*_*LCT*_ is calculated obtaining the liftered cepstrum *CP*_*L*_ *(τ)*;
10. a moving average over a quefrency band of 0.20 s further improves the statistical consistency of *CP*_*L*_ *(τ)*;

Figure 3 shows that liftering removes very-low quefrency power due to noise without affecting the true ECG cepstral peaks. Readers may find a detailed description of the MATLAB code implementing steps from 1 to 10 in the Supplemental Material. We define the quality score as the power of the liftered cepstral peaks relative to the total power of the unliftered cepstrum as follows:

11. the power of *CP*_*L*_ *(τ)* first and second harmonics, *H*, is calculated;
12. the cepstral quality index is the ratio between *H* and *TOT*: CQI = *H/TOT.*

In our application, the main ECG peak is identified between 0.30≤ *τ* ≤ 2.5 s (the quefrency band where *τ*_0_ corresponding to the average R-R interval is expected) by comparison with a threshold equal to the 90% percentile of the liftered cepstrum: the peak of the ECG first cepstral harmonic should overcross this threshold. A second cepstral peak that falls in the quefrency band corresponding to twice the band of the main harmonic is recognized as a genuine second harmonic. CQI may therefore range between 100% (when all the power of the cepstrum is contained in the first and second harmonics of the ECG cepstrum) and 0% (when no ECG peaks are identified in the cepstrum).

When the hypothesis of ECG pseudoperiodicity does not hold, as during arrhythmias episodes, a cepstral main harmonic might not be detected even in a high-quality ECG. Figure 3D shows an ECG segment during atrial fibrillation: the power spectrum does not have a periodic structure, cepstral harmonics cannot be correctly associated with a spectral periodicity, and the resulting CQI is remarkably low.

When the ECG recording is longer than 10 s, continuous, second-by-second CQI estimates are calculated for the whole duration of the recording performing the cepstral analysis on 90%-overlapped, running ECG segments of 10-s length.

### 2.5 Validation on Synthesized and Real Electrocardiograms

We quantified the effects of noise and deviations from pseudoperiodicity on CQI using the synthesized ECGs. The effect of broadband noise was evaluated by adding increasing levels of white noise to the synthesized ECGs, with signal-to-noise ratio, SNR (ratio between the ECG power and the power of added noise), between 9 and 1/9. The effects of deviations from periodicity were evaluated on synthesized ECGs appending the ECG templates at uneven periods. Two types of heart rate changes were simulated preserving the original mean heart rate: monotonic ramps, with R-R intervals increasing or decreasing linearly in time, and random fluctuations, with independent changes of R-R intervals from one beat to the next. Monotonic heart-rate ramps during normal sinus rhythm can be observed in 10-s ECG tracings following cardiac sympathetic or vagal activations, as well as during deep breathing episodes. Random heart-rate changes may somehow model the disordered cardiac rhythm in atrial fibrillation. The variation coefficient, VC (ratio between standard deviation and mean) of the original R-R intervals in healthy volunteers was 4.5% when calculated over 10-s segments and the ECGs were synthesized with VC between half and twice this physiological value. Premature beats may also alter the ECG pseudo-periodicity. Supraventricular beats were simulated by removing the portion of the template before the QRS complex which includes the P wave. One, two, or three altered templates were added randomly to the equispaced sequence of synthesized ECGs.

To validate the proposed cepstral score on real ECG tracings, we considered recordings from two datasets provided by the Physionet Community at the MIT Laboratory for Computational Physiology (Goldberger et al., 2000). To test the effects of noise and artifacts on real ECG recordings, CQI was evaluated on “set-A” of ECG recordings made available for the Physionet/Computing in Cardiology 2011 Challenge (Silva et al., 2011). The recordings consisted of 12 ECG leads (I, II, III, aVR, aVF, aVL, V1-V6) sampled at 500 Hz, 16 bit with 5 μV resolution, and standard diagnostic bandwidth (0.05–100 Hz). The overall quality of each multi-lead recording was manually scored by a group of annotators. Combining their scores, ECG recordings were classified as having “acceptable” (*n* = 773) or “unacceptable” (*n* = 225) signal quality.

To systematically check how the CQI score detects arrhythmias, the cepstral analysis was applied to the training dataset of the PhysioNet/Computing in Cardiology 2017 Challenge (Clifford et al., 2017). The dataset is composed of single-channel (equivalent to lead I) ECGs recorded with the AliveCor devices at 300 Hz and 16 bit, with 0.5–40 Hz bandwidth, visually classified by experts into 4 groups: noisy, or in normal rhythm, or in atrial fibrillation, or in any “other” rhythm. From the whole dataset, we considered the 5050 normal-rhythm recordings, the 738 atrial fibrillation recordings, and the 284 noisy ECGs. Their duration ranged between 9 and 61 s, with a median value of 30 s. The running cepstral analysis was applied and the second-by-second estimates were averaged to obtain a single CQI score for each recording. The Area Under the Curve (AUC) of Receiver-Operator Characteristic (ROC) analysis measured how CQI classifies between cases for which heart rate variability analysis is feasible (i.e., normal rhythm recordings without excessive noise), and cases to be excluded from heart rate variability analysis (i.e., atrial fibrillation or too noisy recordings). The Youden index, calculated as in (Goksuluk et al., 2016), provided the cut-off value for the classification.

Statistical comparisons between groups were performed with the Wilcoxon matched-pairs test.

Additionally, we illustrated the performance of the proposed score by applying the running cepstral analysis on ECG tracings collected in our previous experiments, which included long-term recordings in free-moving volunteers at high altitudes (Lombardi et al., 2013; Caravita et al., 2015). The experiments were approved by the ethic committee of Istituto Auxologico Italiano, IRCCS (EudraCT No. 2010-019986-27) and conducted in agreement with the principles of the Declaration of Helsinki, after having received informed consent.

## 3 Results

### 3.1 Validation on Synthesized Electrocardiograms

**FIGURE 4:**
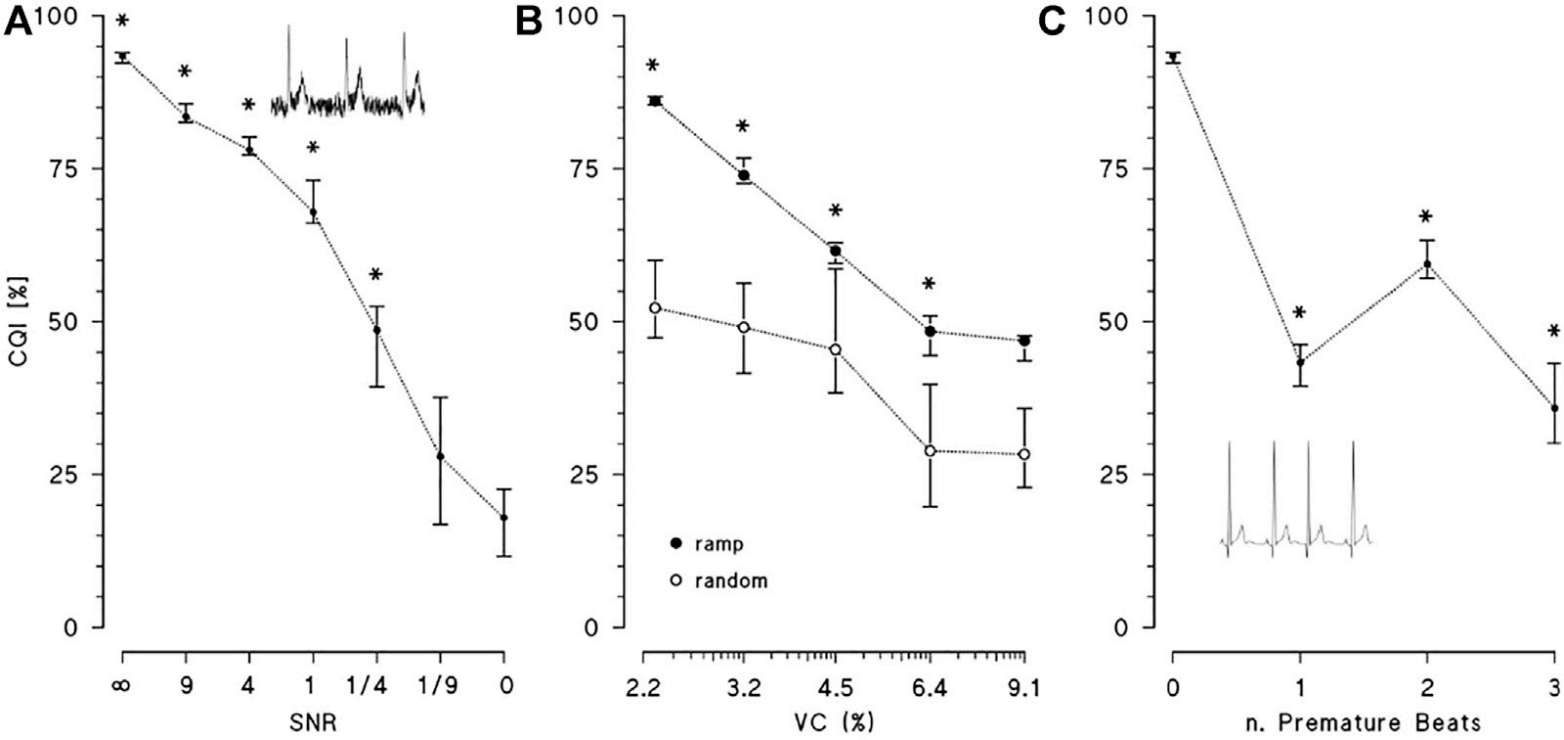
Effects of noise and deviations from periodicity on CQI. CQI median and interquartile range over 8 synthesised ECGs. **(A)**: CQI at decreasing levels of signal-to-noise ratios, SNR (SNR = ∞ means no noise, SNR = 0 means noise only) with * indicating statistically significant difference vs. SNR = 0 at *p* < 0.05; the inset is an example of ECG with SNR = 4. **(B)** *:* CQI at increasing values of variation coefficient, VC, of R-R interval when the heart rate dynamics consists in monotonic ramps (solid circles) or random changes (open circles): median and interquartile range over 8 synthesised ECGs with * indicating statistically significant difference between ramp and random changes at *p* < 0.05. **(C)**: CQI without or with 1, 2 or 3 premature beats, with * indicating statistically significant difference vs. no premature beats at *p* < 0.05: inset is an example of simulated supraventricular premature beat.

Figure 4A shows the effect of broadband noise on CQI. The index progressively decreases from values greater than 90%, when noise is absent, to 68% when SNR = 1. CQI falls more rapidly when SNR < 1 and at SNR = 1/9 it is statistically indistinguishable from CQI of pure noise. Figure 4B illustrates the effects of increasing levels of heart rate variability, quantified by VC, on CQI, comparing random changes with ramp-like changes. In both cases, CQI decreases with increasing levels of VC, but the effects depend on the type of heart rate dynamics, being more important for random than monotonic changes. As to the effects of premature beats on CQI, Figure 4C shows that even a single ectopic beat in the 10-s ECG segment reduces CQI importantly.

### 3.2 Validation on Real Electrocardiograms

#### 3.2.1 Physionet/Computing in Cardiology 2011 Challenge

**FIGURE 5:**
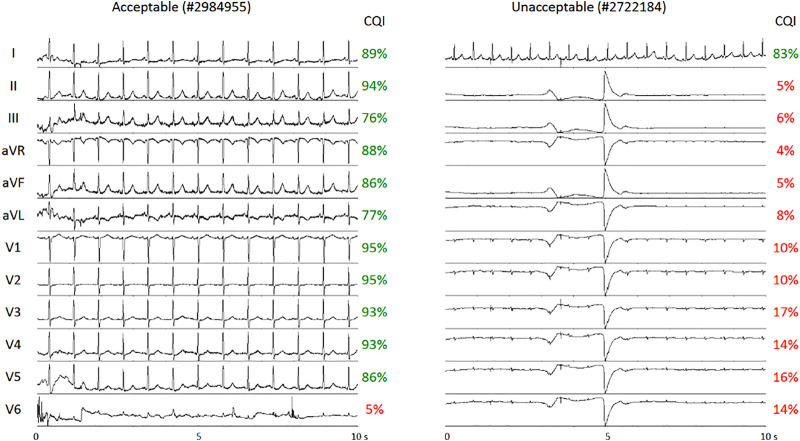
Examples of multi-lead ECGs from the dataset of Physionet/Computing in Cardiology 2011 Challenge. The recordings were classified as “acceptable” (left) or “unacceptable” (right). CQI scores of individual leads correctly detect Lead I of sufficient quality for heart rate variability analysis in the “unacceptable” recording and Lead V6 as inadequate for heart rate variability analysis in the “acceptable” recording.

The effects of noise and artifacts on real ECG recordings were tested systematically on the Physionet/Computing in Cardiology 2011 Challenge dataset (Silva et al., 2011). Figure 5 shows two examples of multi-lead ECGs, one classified as “unacceptable” and one as “acceptable”; the CQI of each lead is reported. Since the Physionet classification regards the quality of multi-lead ECGs as a whole, a single lead might have sufficient quality for heart rate variability analysis even in a multi-lead recording scored as globally unacceptable: this is the case of lead I of #2722184 recording. Similarly, individual leads may occasionally have poor quality even in globally “acceptable” recordings, such as lead V6 of #2984955 recording. In these two examples, the CQI scores allow automatically selecting the proper ECG leads for heart rate variability discarding inadequate leads.

**FIGURE 6:**
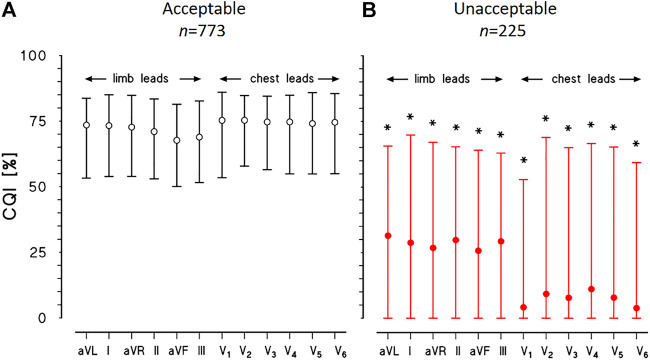
Physionet/Computing in Cardiology Challenge 2011 datasets. CQI median and interquartile range for each of 12 leads of ECG recordings classified as “acceptable” **(A)** or “unacceptable” **(B)**: for each lead, the * indicates statistically significant difference between groups at *p* < 0.01.

Figure 6 shows the results of cepstral analysis for the whole dataset. The median CQI score of each lead is coherent with the manual classification through visual inspection of the recordings, being close to 70% for all the leads of the “acceptable” group and much lower for all the leads of the “unacceptable” group (close to 30% for limb leads, to 7% for chest leads).

However, the CQI interquartile range is remarkably wide for the “unacceptable” group, suggesting that leads with a sufficient CQI score for heart rate variability analysis may be identified in most cases even in this group, as the example of Figure 5A suggests.

#### 3.2.2 Physionet/Computing in Cardiology 2017 Challenge

**FIGURE 7:**
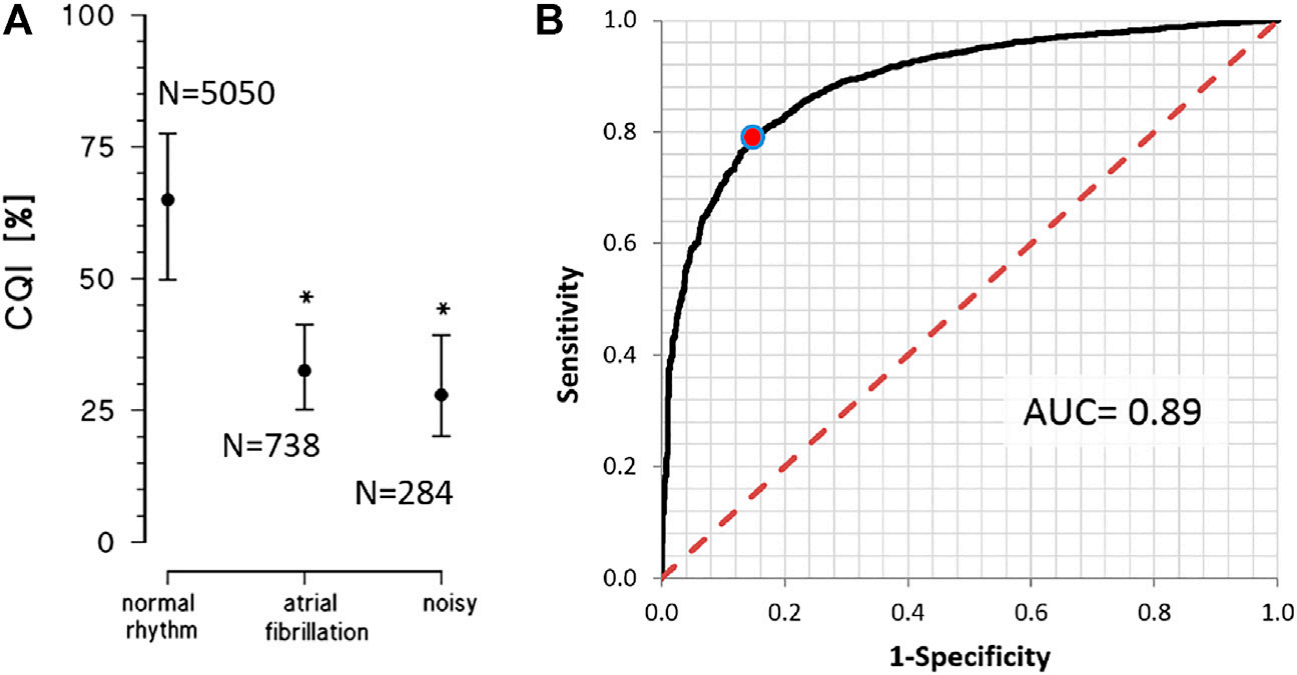
Physionet/Computing in Cardiology Challenge 2017 datasets. **(A)** CQI as median and interquartile range for single-lead ECGs manually classified as in normal rhythm, atrial fibrillation or noisy recording. The * indicates a statistically significant difference vs. the normal rhythm group at *p* < 0.01 **(B)** ROC analysis on 5050 recordings adequate for heart rate variability analysis (normal rhythm) vs. 1022 recordings inadequate for heart rate variability analysis (in atrial fibrillation or noisy); the red dot on the curve identifies the classification cut-off (CQI > 47%) according to the Youden’s criterion; AUC = Area Under the Curve.

Figure 7A compares CQI values in the three groups of recordings of the PhysioNet/Computing in Cardiology 2017 Challenge (Clifford et al., 2017). These three groups were selected to quantify the effects of deviations due to noise or atrial fibrillation from the pseudoperiodicity of the normal rhythm. Most of the recordings in normal rhythm have CQIs greater than 50%, as the “acceptable” recordings of the 2011 Challenge. Atrial fibrillation or noise substantially reduces CQI, which is lower than 40% in most of these recordings.

The ROC curve that was calculated to quantify the capability of CQI to identify an ECG recording as adequate for heart rate variability analysis, was associated with a consistently high AUC (Figure 7B). The Youden criterion identified in CQI > 47% the cut-off to classify an ECG recording as adequate, with 79.0% sensitivity and 85.2% specificity.

#### 3.2.3 Running Cepstral Analysis

**FIGURE 8:**
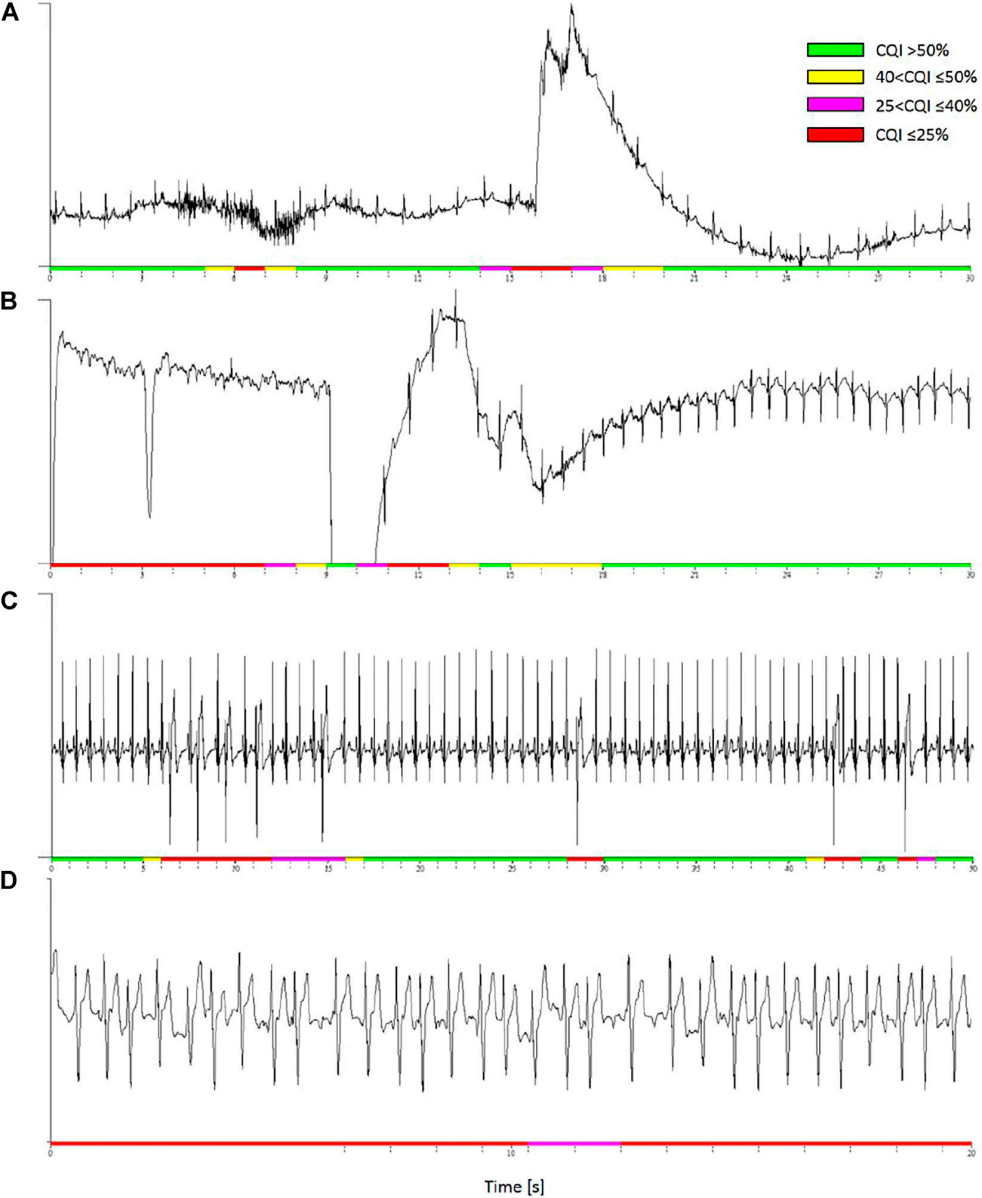
Examples of discarded ECG segments by running cepstral analysis. Colors represent four CQI levels. **(A)** *:* low CQI values identify a short burst of muscle noise and a following movement artefact. **(B)** *:* low CQI values detect signal loss likely due to a poor contact between textile electrodes and skin. **(C)**: premature beats in a high quality recording are locally associated to low CQI values. **(D)** *:* atrial fibrillation is associated to persistently low CQI even in high quality ECG recording.

Figure 8 shows examples of running cepstral analysis on ECGs collected with wearable/mobile devices affected by different types of noise. A four-level color code is used to represent the CQI values estimated second by second. Based on the cut-off defined by ROC analysis (Figure 7B), we classified the ECG as having good quality when CQI > 50%, representing the time window in green color, and as having “acceptable quality” when 40 < CQI ≤ 50%, representing the time window in yellow. ECG classified with “very low” or “unacceptable” quality are associated with 25 < CQI ≤ 40% and CQI ≤ 25%, and are represented in magenta and red respectively.

Panel a) is a segment of 24-h ECG (250 Hz sampling, 16 bit) in a healthy volunteer during daily-life activities with a wearable device (Faros 360 Mega, Kuopio, Finland). The ECG shows a short burst of muscular noise followed by a movement artifact: the running analysis associates both the events with locally low CQI values.

Panel b) is a segment of ECG recorded in a healthy volunteer during night-time sleep at a high altitude (Lombardi et al., 2013). The ECG (200 Hz, 12 bit) was recorded with the MagIC device, a wearable system with woven ECG electrodes, a textile plethysmograph for measuring respiratory movements of the thorax, and a sternal accelerometer (Di Rienzo et al., 2010), connected to a pulse oximeter (Nonin Xpod^®^, Nonin Medical, Inc., Plymouth, MN, United States). The running analysis classified unacceptable (CQI ≤ 25%) a data segment with a temporary signal loss, likely due to a bad contact between textile electrodes and skin. The recovery of the ECG waveform was identified by classifying the signal as having CQI > 50%.

Panel c) is a high-quality ECG with frequent premature beats recorded by MagIC in a volunteer resting at a high altitude (Caravita et al., 2015). While most of the recording is associated with high CQI values, each premature beat causes a dramatic local fall in the quality score. These beats are classified as “unacceptable” for heart rate variability analysis.

Panel d) is an example of running cepstral analysis during random heart-rate variations due to the lack of normal sinus rhythm. The ECG was recorded with a mobile electrocardiograph (AliveCor Inc., Mountain View, CA, United States) by a patient in atrial fibrillation (#A00027 of the PhysioNet/Computing in Cardiology Challenge 2017 dataset). The whole signal is associated with very low CQI values indicating that it is unacceptable for heart rate variability.

**FIGURE 9:**
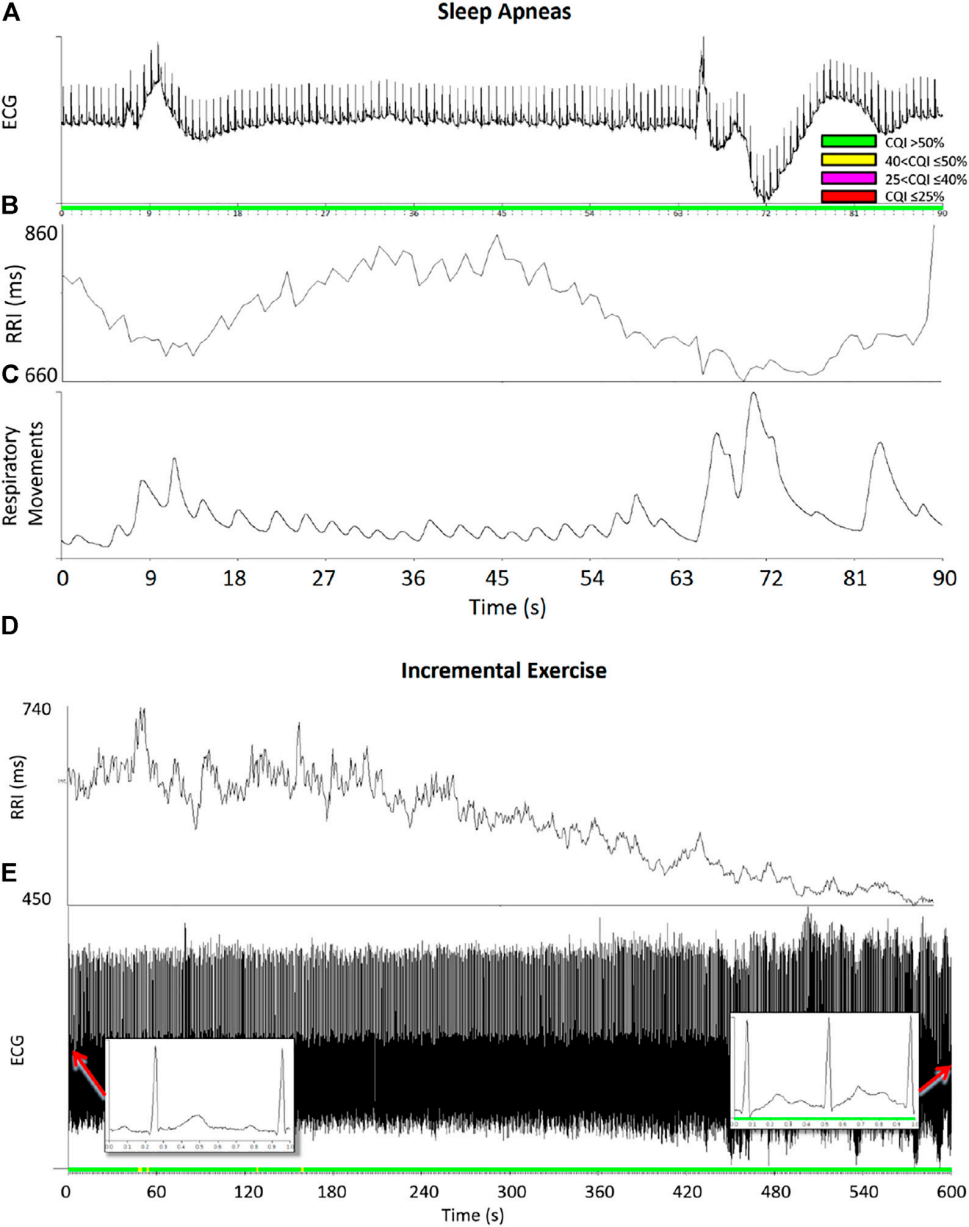
Examples of running cepstral during ramp-like heart-rate changes. Upper panels: ECG **(A)**, R-R intervals, RRI **(B)**, and respiratory movements of the thorax **(C)** during sleep apneas; color codes represent the CQI levels. Even if apnea/deep breathing events produced large ramp-like changes of heart rate, the CQI score remained relatively high classifying the data segment of good quality for heart rate variability analysis. Lower panels: R-R intervals **(D)** and ECG **(E)** during an incremental exercise test on the cycloergometer: the ECG was in normal sinus rhythm (see insets) and the CQI score remained high even when RRI progressively decreased during the exercise.

Figure 9 shows examples of running cepstral analysis on ECG tracings in normal sinus rhythm with large ramp-like changes in heart rate. The upper panels regard a segment of a sleep recording at a high altitude by the MagIC device. The low barometric pressure at high altitude induced frequent apneas/hypopneas events followed by deep breathing, which caused wide ramp-like changes in R-R intervals and fluctuations of the ECG baseline. These wide heart rate fluctuations occurred in normal sinus rhythm and did not prevent the cepstral analysis to quantify high CQI scores and classify the segment as acceptable for heart rate variability analysis.

The lower panels of Figure 9 regard an ECG recording (1000 Hz sampling rate, PowerLab 8/35 Bioamp—Data Acquisition System, ADInstruments, Dunedin, New Zealand) during an incremental exercise test up to exhaustion on the cycle ergometer (Ergoselect 100, ergoline GmbH, Bitz, Germany). The test was performed by a 14-year-old male participant with an increasing exercise load at 12 W/min. Even if the R-R intervals decreased progressively during the test with a ramp-like pattern as the load increased, the CQI score remained sufficiently high and classified the ECG quality as “good” or “acceptable” throughout the test.

## 4 Discussion and Conclusion

In the last years, several methods have been proposed for evaluating the quality of ECG recordings: the principles on which they are defined and comparisons among methods are reported in two recent reviews (Satija et al., 2018; Rahman et al., 2022). These reviews highlight that the accuracy of each method depends on the medical context for which it is proposed. Therefore, while some methods are aimed at the correct identification of the QRS complex only, others require a more detailed morphological identification of specific ECG features, like amplitudes and intervals between waves (Satija et al., 2018). Furthermore, the accuracy depends on the testing dataset because the type of artifacts and noises affecting mobile ECGs for telemonitoring applications differ from those expected for wearable ECG devices or ECGs recorded in the doctor’s office or in intensive care units (Rahman et al., 2022). In this context, the usefulness of our cepstral approach is to address a specific aspect of the ECG signal quality that has not been explicitly considered by other methods: the acceptability of ECG recordings for heart rate variability analysis. In this frame, an ECG segment should be considered not acceptable even in absence of noise components or artifacts if it does not occur during normal sinus rhythm, a condition not considered by other indexes of signal quality. In addition, our work was motivated by the need to evaluate the ECG adequacy for heart rate variability analysis continuously onboard wearable devices with low computational power. We found the cepstral analysis to be a promising approach because the power cepstrum describes the periodic structure of ECG recordings in a simple way (i.e., with the main harmonic at *τ*_0_ and very few multiples at most) that can be calculated and interpreted easily. This makes the power cepstrum a potentially useful tool in heart rate variability studies to identify and discard noisy ECG recordings or recordings not in normal sinus rhythm. The estimation of the power cepstrum does not require important computational resources and consent defining a simple score, CQI, evaluable by the wearable systems themselves. In multi-lead ECG recordings, CQI could allow selecting and transmitting the ECG lead with the best quality only (that is the lead with the highest CQI), reducing the flow of redundant information within the monitoring system and the power consumption for signal transmission.

**FIGURE 10:**
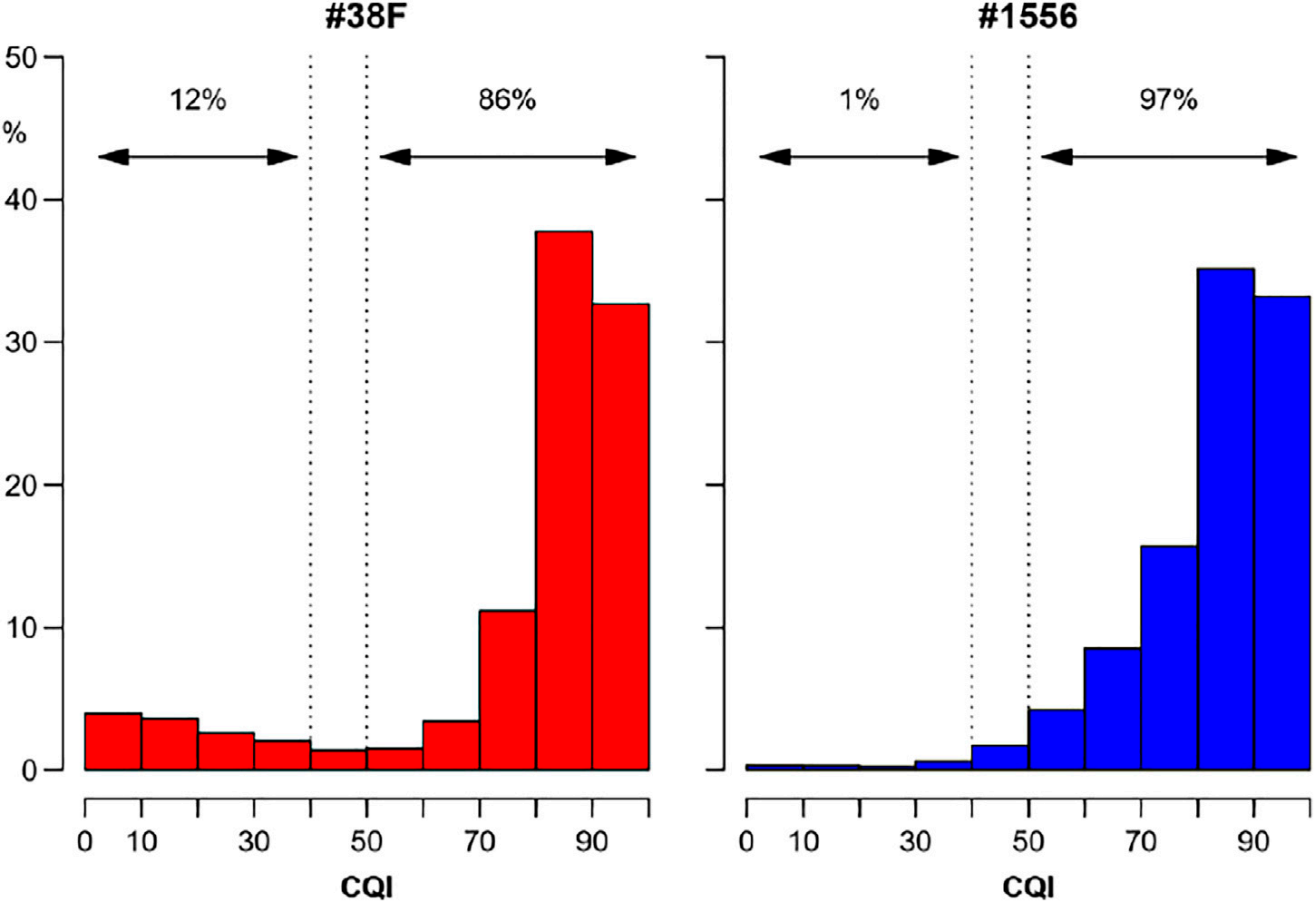
Overall quality of very long-term ECG recordings from the distribution of running CQI values. Distributions (relative frequencies) of CQIs calculated second-by-second on ECG Holters recorded for 7 consecutive days continuously with a wearable device (RootiRx^®^, Rooti Labs Ltd., Taipei, Taiwan) at 250 Hz in two subjects. The recording of the subject on the right (#1556) is almost completely analysable having good quality (CQI > 50%) for 97% of the time; by contrast, assessing heart rate variability in the recording on the left (#38F) might be problematic for a not negligible fraction of the time, being the quality “very low” or “unacceptable” (CQI ≤ 40%) for 12% of the recording.

The dataset of the Physionet/Computing in Cardiology 2017 Challenge demonstrated that CQI is useful to distinguish ECGs in normal rhythm from unacceptable recordings due to noise or arrhythmias, and provided us with an objective cut-off threshold for the classification. In ambient-assisted living applications, thresholds on running CQI estimates may be employed to send alerts from the wearable device to upper computational levels, which may apply more sophisticated analysis tools, possibly integrating other physiological, behavioral, and environmental signals, to properly manage the alarm. On very-long term monitoring, comparing the distribution of CQI values with cut-off thresholds provides an effective way to summarize the overall quality of the ECG recording, as in the example of Figure 10.

It is worth noting that deviations from the ECG periodicity may occur also in normal sinus rhythm due to physiological changes in heart rate. Even if most applications of heart rate variability require stationarity, which assures a stable mean heart rate, time-varying methods are employed to describe autonomic activations that induce rapid changes in the cardiac rhythm. These changes appear as heart-rate ramps in the short running window used for cepstral analysis and they should not be excluded from the evaluation of heart rate variability. Results on synthesized ECGs (Figure 4B) showed that a chaotic heart-rate pattern decreases CQI significantly more than a ramp-like pattern with the same variation coefficient. As Figure 9 exemplifies, this means that even marked ramp-like changes in the heart rate may be correctly classified as adequate for evaluating the heart rate variability if they occur in normal sinus rhythm, in absence of artifacts or noise.

### 4.1 Limitations and Future Perspectives

We defined CQI to assess recordings in adults or the elderly during daily activities. Therefore, the parameters defining our method should be modified to properly monitor subjects with much higher heart rates, such as neonates or young athletes during maximal exercise. This can be done easily because the parameters are easily interpretable. For instance, let’s consider the frequency data window we defined between 0 and 20 Hz and the cepstral band for identifying the peak at *τ*_0_ between 0.3 and 2.5 s; if the heart rate is 180 bpm, only 6 harmonics fall in the frequency data window and *τ*_0_ is very close to the lower limit of the cepstral band. Thus, if such high average heart rates are expected, it may be desirable to increase the upper limit of the frequency window above 20 Hz and to shift the cepstral band toward quefrencies lower than 0.3 s to better capture the cepstral power around the mean R-R interval. Moreover, a limit of CQI is that it does not distinguish between noise and arrhythmias, being similarly low in the case of noise and atrial fibrillation (Figure 7A), even if the causes for the deviations from the ECG periodicity are rather different in the two cases. More detailed quantification of the cepstral morphology than CQI might better characterize the chaotic rhythm of atrial fibrillation, possibly integrating traditional spectral methods to distinguish among types of atrial fibrillation and between atrial fibrillation and noise.

## Data Availability

Publicly available datasets were analyzed in this study. This data can be found here: Physionet https://physionet.org/about/challenge/moody-challenge. The Matlab code described in the Supplemental Material can be accessed at doi:10.5281/zenodo.6552328.
