# Supplementary material for: Cepstral Analysis for Scoring the Quality of Electrocardiograms for Heart Rate Variability
Source: Front Physiol. 2022 Jun 17;13:921210. doi: 10.3389/fphys.2022.921210 (PMC9247307; doi:10.3389/fphys.2022.921210)
Supplement: Supplementary file 1 [file DataSheet1.PDF]

Supplementary Material

1 Matlab code to calculate the ECG Cepstrum

The software used in our analysis was designed to run onboard the microcontroller of our wearable system (MagIC system) and was therefore compiled for better performance. We provide a Matlab translation of our cepstral ECG estimation code, splitting and commenting the code into blocks referring to steps 1 through 10 in section 2.4. The Matlab function "cp.m" and examples of its outputs can be downloaded from the Zenodo repository at doi: 10.5281/zenodo.6552328

Given a time series ts of length N with sampling frequency fs, ts is linearly detrended and Blackman windowed (see 2.4 point 1).

CODE 1:

```
N = length(ts);

% Linear detrending
ts = detrend(ts, 1);

% Blackman window
w = blackman(N);
s = ts.*w;
```

The FFT power spectrum of ts is calculated from 0 to Nyquist frequency, fs/2, with zero-padding to the next higher power of 2 (see 2.4 point 2). With a frequency spacing between samples of fs/N Hz, the sampling quefrency is τ= N/fs s.

CODE 2:

```
N = pow2(ceil(log2(N))); % Number of points for zero-padding
ffts = fft(s, N);
ffts = ffts(1:N/2+1);
P = (1/(fs*N)) * abs(ffts).^2;
P(2:end-1) = 2*P(2:end-1);
freq = 0:fs/N:fs/2;
tau = N/fs; % Sampling quefrency
```

The FFT spectrum is smoothed with a moving average, where each mean is calculated over a sliding window of length 0.3 Hz across all neighboring elements, and log-transformed (logP). The logP is limited between 0 and 20 Hz (see 2.4 point 3).

Code 3:

```
wndFFT = 0.3; % in Hz
fmaxCepstrum = 20; % in Hz

% Moving average and log-transformation
step = round(wndFFT/freq(2) + 1);
P = movmean(P, step);
logP = log10(P);

% logP is limited between 0 and fmaxCepstrum
logP = logP(freq <= fmaxCepstrum);
```

The logP spectrum is detrended removing the kth-degree polynomial trend, and windowed with a 10%-cosine taper. The order of polynomial trend is 1 for the original cepstrum (see 2.4 point 4) or 10 for the liftered cepstrum (see 2.4 points 7-8).

Code 4:

```
k = 1; % or 10

% Removes the detrendFFTCepstrum-degree polynomial trend
logP = detrend(logP, k);

% 10%-cosine tapper
w = tukeywin(length(logP),0.1);
logP = logP.*w;
```

The FFT power spectrum of logP is calculated from 0 to Nyquist quefrency, τ/2, with zero-padding to the next higher power of 2 (see 2.4 points 5 and 9).

Code 5:

```
N = length(logP);
N = pow2(ceil(log2(N))); % Number of points for zero-padding
fftlogP = fft(logP,N);
fftlogP = fftlogP(1:N/2+1);
CP = (1/(fmaxCepstrum*N))*abs(fftlogP).^2;
CP(2:end-1) = 2*CP(2:end-1);
quef = 0:tau/N:tau/2;
```

The CP is smoothed with a moving average over a sliding window of length 0.2 s (see 2.4 point 10).

Code 6:

```
wndCepstrum = 0.2; % in seconds

% Moving average
step = round(wndCepstrum/quef(2) + 1);
CP = movmean(CP,step);
```

The cepstrum estimate is returned at quefrencies ≥ 0.05 s (1/20 Hz, see code 4).

Code 7:

```
CP = CP(quef>=1/fmaxCepstrum);
quef = quef(quef>=1/fmaxCepstrum);
```
